# Japanese encephalitis: Challenges and intervention opportunities in Nepal

**DOI:** 10.14202/vetworld.2015.61-65

**Published:** 2015-01-17

**Authors:** Shristi Ghimire, Santosh Dhakal

**Affiliations:** 1 National Zoonoses and Food Hygiene Research Center, Kathmandu, Nepal; 2 Department of Veterinary Preventive Medicine, Food Animal Health Research Program, Ohio Agricultural Research and Development Center, The Ohio State University, Wooster, Ohio, USA

**Keywords:** geographical expansion, Japanese encephalitis, Japanese encephalitis virus, Nepal, pig

## Abstract

Japanese encephalitis (JE) is a mosquito borne zoonotic disease caused by JE virus (JEV). JE has been endemic in Terai region, the lowland plains of Nepal bordering India, since 1978. However, in recent years cases of JE has been continuously reported from high altitude zones of hills and mountains. Irrigated rice farming system, expanded pig husbandry practices, inadequate vaccine coverage, low level of public awareness and climate change favoring mosquito breeding in higher altitudes might be the probable risk factors for emergence and re-emergence of JE in Nepal. Repeated outbreak in endemic areas and geographical expansion to newer areas have created huge challenge for JE prevention and control. At present, JE is one of the major public health concern of Nepal. Expanding vaccine coverage, improving agricultural practices, generating public awareness, supporting for use of mosquito avoiding practices and regional collaboration at border against JE can be helpful in getting better control over it in future.

## Introduction

World Health Organization has quoted “a person of any age, at any time of the year, with the acute onset of fever and a change in mental status or new onset seizures (excluding simple febrile seizures)” as the basis for defining acute encephalitis syndrome (AES) cases [1]. Japanese encephalitis (JE) is the AES case that is confirmed by detection of JE virus specific antibody in a single sample of cerebrospinal fluid (CSF) or serum [2]. It is the major cause of encephalitis in Asia with annual incidence of around 45,000 cases and 10,000 deaths [3]. However, because of insufficient medical facilities and inadequate data collection in developing countries, JE cases are underreported and cases are estimated to be even higher [4]. Ardeid wading birds are the primary enzootic hosts of JE virus (JEV). Pigs are the major amplying host and mosquitoes of *Culex vishnui* subgroup and particularly *Culex tritaeniorhynchus*, that breeds predominantly in paddy field, are the major vector responsible for JEV transmission. Human beings are the dead end hosts [3].

JEV mostly affects children and young adults [5]. Confirmation of JE is done only after serological examination and positivity for specific anti-JE IgM in the CSF or serum at the time of illness [6]. Infection in human produces wide range of clinical manifestations ranging from asymptomatic infection, through mild febrile illness, to acute and lethal meningo-myeloencephalitis. One third of the clinical cases are fatal while half of the survivors develop neurological sequelae [7]. Like other viral disease, JE also has no specific treatment and only supportive treatments are given [6].

## JE World Scenario

Origin of JEV is traced back to Malay Archipelago through genetic studies [8]. JEV was isolated from human case in 1934 and in 1938 the role of *C. tritaeniorhynchus* as a vector, and wading ardeid birds and pigs as reservoir hosts was known [9,10]. Major epidemics were reported from Japan (1871 and 1924); northern Vietnam (1965); Thailand (1969, 1970); India (1973); Nepal (1978) and from Sri Lanka (1985-87) [11-13]. At present, the geographic range of JEV infection extends from eastern to Southeast Asia and northern Australia, and to southern Asia [3]. However, it is likely to increase in Bangladesh, Cambodia, Indonesia, Laos, Myanmar, North Korea, Pakistan, Philippines and other countries because of population growth, intensified rice farming, pig rearing, and the lack of vaccination programs and surveillance [8].

### Risk Factors for JE

JE is spread by marsh birds through *Culex* mosquitoes and intensified by pigs where humans are dead end hosts. JE transmission cycle is complex involving various ecological, environmental, climatic and human behavioral factors [14]. Rice farming, pig rearing and rural population are the contextual risk factors for JE [15,16]. *C. tritaeniorhynchus*, the primary vector of JEV is found mainly in irrigated rice fields and JE is concentrated mainly in countries where extensive irrigated rice agriculture system exists [5,15-17]. In India, where irrigated rice agriculture system is extensively developed, occurrence of JE was closely associated with high vector densities either breeding in the rice fields or canal system [18].

Pig is the major amplifying host for JEV and prolonged viremia occurs in pigs. Hence, pig rearing is also a risk factor for JE. Pigs are often common in endemic areas and maintained without proper sanitary and hygienic considerations [7,18]. Proximity to rice fields, pig ownership and older age are recorded as the risk factors for JE in Indonesia [15]. High proportion of rice planting, rural population and extent of pig rearing were regarded as the contextual risk factors of JE in China [16]. Poverty is the insufficient indicator of the occurrence and pattern of JE [19]. However, poor people who have less access to health facilities are likely to be affected more frequently and severely. The poorest sectors of the population like pig farmers are at higher risk when they sleep outside during hot humid vector dominating months and often sleep close to the pigs. For example, in Central China, more JE cases were observed among children living in poor quality houses and whose parents had lower income [20]. Human and pig vaccination are regarded as the reliable method of preventing JE [3].

## History and Characteristics of JE in Nepal

Epidemics of JEV travelled northward in India and began to appear in Nepal in the late 1970s. The first clinical diagnosis of JEV infection was done in 1978 [21]. Since then, JE has been endemic in Terai region of Nepal. Terai is a tropical, low land plains of Nepal bordering India which is experiencing larger outbreaks in every 2-5 years. High temperature, high humidity, vast rice fields and flourishing pig production are responsible for endemicity in Terai region [22,23]. A total 26,667 cases and 5,381 deaths occurred from JE in Nepal within the 25 year period (from 1978 to 2003) with 20.2% average case fatality rate [6]. Though it is mainly a disease of children, JE has been confirmed in all age groups in Nepal and higher cases reported in males than in females [24]. JE has a typical seasonal pattern of outbreak in Nepal. Though cases are reported throughout the year, upsurge takes place after the rainy season, which supports for rice plantation and hence mosquito breeding. Incidence is highest between July to September each year and peaks during August [6,24].

### Present status of JE in Nepal

Today’s main concern about JE in Nepal is its geographical expansion to high altitude. Zimmerman *et al*. reported the first proven cases from Kathmandu valley, which is a hill region (1300 masl) [25]. Patridge *et al*. first studied about the endemicity of JE in Kathmandu valley. In their study, they followed up the all lab confirmed cases of JE to collect information of residency and travel history and found maximum cases had no history of travel outside the valley 30 days before onset of illness. Now, JE has been documented to be endemic in the Kathmandu Valley as well as other 24 districts and cases have been detected in a total of 54 out of 75 districts in Nepal [24,26].

These days even the mountainous regions are reporting JE. From 2004 through 2006, a total of 108 laboratory confirmed JE cases were reported from hill and mountain districts excluding Kathmandu valley [27]. Thakur *et al*. conducted a cross-sectional survey from July to August of 2010 in four mountain districts (Sindhupalchowk, Dolakha, Solukhumbu and Kavrepalanchowk) known to have human cases of JE. A total of 454 pig serum samples were collected and tested by competitive ELISA. Results showed that 16.7% (76/454), of pigs had anti-JEV antibodies suggesting for probable endemicity of JE in these districts [28]. Conventional logic does not make the high altitude zones be vulnerable for vector borne diseases like JE. However, the situation being faced in Nepal is totally different. Year after year, cases of JE are reported from hills and mountain region. JE cases with no travel history of patients to JE endemic regions before illness suggests for probable endemicity of JE in different ecological system of Nepal. It has been suggested that the shifts in JE case distribution may be due to conferred widespread immunity in Terai resulting fewer susceptible people due to immunization. Mass immunization campaigns introduced in 2006 is being conducted in phase wise manner starting from highest priority districts [26]. Climate change and adaptation of vector to hitherto cold climate and growing pig farming practices in high altitude areas can be the other supporting factors for JE geographical expansion in Nepal [29].

## Challenges in JE Prevention and Control in Nepal

JE has been a major public health concern in Nepal owing to its emergence in new area and persistence in endemic region with high mortality and disability rates. However, the complex transmission pattern of JEV is yet to be fully understood in this country. Various challenges for JE prevention and control include - (i) land use pattern and rice cultivation - paddy is not only the staple food, but also is a major economic activity and key source of employment and income generation in Nepal. Paddy farming is carried out all over the country. In Nepal a stronger association is found to exist between JE incidence and percentage of irrigated land, mainly the paddy field. However, sudden improvements in agricultural practices is not feasible [29,30] (ii) growing pig husbandry without sanitary and hygienic consideration: Government of Nepal has been prioritizing pig farming as the potential means of alleviating poverty. The small investment and relatively higher return in pig farming has attracted all class of people in this sector. The farms are however, maintained under unsanitary practices [30]. Rao’s proposal of 5 kilometre distance between household and pig farming for JE control is also not feasible in Nepal because of peri-urban migration; small pig population; farming not on own land and mobile population, which directly affect on policy making to set distances between people and pigs [31,32]. (iii) Climate change and vector adaptation to high altitude region (iv) Close pig-human-rice field interaction (v) poverty and access to health care: like other tropical disease, JE also disproportionately affects the poor more frequently and more severely. (vi) lack of awareness and prevention practices: Though the disease is existing for long, people are still not much aware about the causes, consequences and prevention practices of the disease. Even in some areas where vaccination is already started by government, the high risk groups like pig farmers are not getting vaccinated. This might be due to unwillingness or being unaware of the vaccination program. Mosquito avoidance practices are also not adequately used (vii) lack of clarity and feasibility on vaccination issues: Government has started human vaccination in phase wise manner in different endemic districts. However, many major issues like the length of immunity provided by vaccine, targeting only children or all age groups for vaccination, conducting vaccination only at endemic areas or whole country etc. are still unanswered [29,32].

### JE intervention opportunities in Nepal

SEARO/WHO has pointed out four strategies for JE prevention and control in countries including Nepal. Those points include (i) health education (about JE and preventive measures); (ii) Vector control (short term measures like insecticide spraying or larviciding activities to long term activities like water management, selection of rice varieties that need minimum water etc.); (iii) Immunization (for human and pigs); and (iv) epidemic preparedness and response [33]. Countries like Japan, Taiwan and South Korea where JE had been a huge problem before have been succeeded to get cases at minimum level these days [8,34]. The following key control strategies and developments might explain the successful decline of JE in these countries: (1) Large scale human immunization program, (2) pig immunization and the separation of pig rearing from human settlements, (3) changes in agricultural practices (e.g. enhanced mechanization and decrease of irrigated land), and (4) improved living standards (e.g. better housing and urbanization) [8]. The high cost involving methods, however, are almost unlikely for Nepal [29]. In Nepal vaccination campaign was started in phase wise manner from 2006. By, 2009, campaigns had been implemented in 23 districts of 27 high and moderate risk districts. In 11 districts children from 1 to 15 years are targeted while in rest 12 districts whole population 1 year or above were vaccinated [30]. Live attenuated SA-14-14-2 vaccine was used (as in previous mass campaigns in Nepal) and the vaccination has reduced cases significantly in coverage areas. There is need of expanding the coverage area of ongoing vaccination [29,30].

Regular use of mosquito avoiding practices can be another low cost JE prevention method. A population based case control study in China, had revealed great reduction in JE cases in population using insecticide treated nets [35]. SEARO-WHO quoted “health education and training is an important part of JE control” [33]. Houston and Chhetry also noted the lack of awareness being potential risk factor of JE in Nepal [36]. Thus, there is need of generating public awareness about the diseases, its transmission pattern, consequences and preventive measures. This can be done through media, health sectors or even by including information about this kind of diseases in school education. Community leaders or professional leaders like village level animal health workers can be the trusted source for dissemination of health information. Besides these attempts should be made to improve agricultural practices mainly the rice farming pattern and pig husbandry. Pig houses should be separated from human dwellings and should follow basic hygienic and sanitary standards. Further problem based research must be carried out to ascertain the complex transmission dynamics of disease in the country [29,32].

## Conclusion

The mosquito borne zoonotic disease JE is a major public health concern in Nepal. Many other countries where JE was a great threat previously have got control over it by effective vaccination strategies for pig and human, and improvements in agricultural practices. However, JEV is expanding its geographical range in Nepal each year. Inadequate vaccination coverage in human; no vaccination in pig; low level of public awareness; inadequate use of mosquito avoiding practices; climate change with expansion of vector into new locality; growing pig husbandry without sanitary and hygienic consideration and existing irrigated rice farming agriculture system can be the factors responsible for emergence and re-emergence of JE in Nepal. In order to get control over it, Nepal government should (i) expand JE vaccine coverage to new areas where this disease has been occurring; (ii) increase public awareness about the cause, consequences, and prevention practices of JE by efficient use of media, health sectors, school teaching or community intervention; (iii) encourage and support for adequate use of mosquito avoiding practices like use of mosquito nets; (iv) give high priority on improved agricultural practices like irrigation system management and pig husbandry practices and (v) work in collaboration with India at border region to control cross country transmission of disease. Nepal needs to address these issues and formulate a short, medium and long-term strategies to get better control over JE.

## Author’s Contribution

S. Ghimire and S. Dhakal generated the concept, collected materials, and wrote the article.
